# Dietary fiber, organic acids and minerals in selected wild edible fruits of Mozambique

**DOI:** 10.1186/2193-1801-2-88

**Published:** 2013-03-08

**Authors:** Telma Magaia, Amália Uamusse, Ingegerd Sjöholm, Kerstin Skog

**Affiliations:** 1 Department of Biological Science, Science Faculty, Eduardo Mondlane University, PO Box 257, Maputo, Mozambique; 2 Department of Chemistry, Science Faculty, Eduardo Mondlane University, PO Box 257, Maputo, Mozambique; 3 Department of Food Technology, Engineering and Nutrition, Lund University, PO Box 124, Lund, SE-221 00 Sweden

**Keywords:** Wild fruits, Minerals, Citric acid, Dietary fiber, Daily intake

## Abstract

The harvesting, utilization and marketing of indigenous fruits and nuts have been central to the livelihoods of the majority of rural communities in African countries. In this study we report on the content of dietary fiber, minerals and selected organic acids in the pulps and kernels of the wild fruits most commonly consumed in southern Mozambique. The content of soluble fiber in the pulps ranged from 4.3 to 65.6 g/100 g and insoluble fiber from 2.6 to 45.8 g/100 g. In the kernels the content of soluble fiber ranged from 8.4 to 42.6 g/100 g and insoluble fiber from 14.7 to 20.9 g/100 g. Citric acid was found in all fruits up to 25.7 g/kg. The kernels of *Adansonia digitata* and *Sclerocarya birrea* were shown to be rich in calcium, iron, magnesium and zinc. The data may be useful in selecting wild fruit species appropriate for incorporation into diets.

## Background

Fruits are generally recognized as essential for health optimisation, with human health depending to a large extent on factors such as high fruit and vegetable consumption (Ibrahim 2011). Deficiencies of essential micronutrients found in fruits can increase the risk of illness or death from infectious diseases by reducing immune and non-immune defenses and by compromising normal physiology and development (Black 2003). Such nutrient deficiencies are highly prevalent in low and middle income countries.

Recent research has shown that a wide range of indigenous fruit trees have the potential to provide rural households with a means to meet their nutritional and medicinal needs (Ekesa et al. 2009). In the past decades several reports have been published on the nutritional composition of wild fruits and vegetables growing in different areas in various African countries and on the effect they could have on combating malnutrition and poverty in the continent (FAO 2011). In June 2008, the European Commission authorised the placing on the market of dried pulp of one wild fruit, Baobab (*Adansonia digitata*), as a novel food ingredient (Commission Decision 2008). Three components are of special importance for determining whether such ingredients are health-enhancing: the kind and amount of minerals, organic acids and dietary fiber.

Minerals are of great importance in the diet, although they comprise only 4–6% of human bodyweight. Some minerals or macro elements required in amounts greater than 100 mg per day represent 1% or less of bodyweight (Insel et al. 2011; Imelouane et al. 2011). The essential macro elements include calcium, phosphors, magnesium, potassium, sodium, sulfur and chloride. Essential trace elements such as zinc, iron, copper, manganese, selenium, iodine and molybdenum are normally required in amounts of less than 100 mg per day, making up less than 0.01% of the bodyweight (Imelouane et al. 2011).

Dietary fiber is increasingly viewed as an essential aspect of good nutrition. Intake of dietary fiber alters the water content, viscosity, and microbial mass of the intestinal contents, resulting in changes in the rate and ease of passage through the intestine (Elleuch et al. 2011). High intake of dietary fiber plays a significant role in weight control and the prevention of several diseases. For example, dietary fiber improves glucose tolerance, by delaying the transport of carbohydrates into the small intestine, reducing the risk of heart diseases and reduces constipation (Anderson et al. 1994; Rodríguez et al. 2006). Organic acids are involved in human growth, maturation and senescence (Al-Farsi et al. 2005). The organic acids influence organoleptic properties such as flavor, color and aroma and are responsible for many characteristic fruity tastes. They increase shelf life, stability and microbiologic safety (Hasib et al. 2002; Loredana et al. 2006; Nour et al. 2010).

In a previous study we reported on the proximate composition of five wild fruits of Mozambique. *Adansonia digitata, Landolphia kirkii, Salacia kraussii, Sclerocarya birrea* and *Vangueria infausta* were evaluated for pH and titratable acidity and their content of dry matter, fat, protein, ash, soluble solids and sugar content (Magaia et al. submitted). The present study was carried out to further analyze the nutritional potential of these fruits. The dietary fiber, organic acids and mineral content of the five wild fruits and selected seeds were determined with the plan to highlight their potential as an effective means to combat macro and micronutrient deficiencies, especially in children.

## Results

The contents of insoluble and soluble dietary fiber in the fruits are presented in Table 1. All pulps and kernels contained dietary fiber, but with large variations in concentration among the different fruits. In *S. kraussii* and in *V. infausta*, the content of insoluble dietary fiber ranged from 2.6 g/100 g to 45.8 g/100 g in the pulps, and from 14.7 to 20.9 g/100 g in the kernels, respectively. The content of insoluble dietary fiber in *A. digitata* pulp was significantly higher than that in *L. kirkii* and *S. kraussii*. The soluble dietary fiber in the pulps ranged from 4.3 g/100 g in *L. kirkii* up to 65.6 g/100 g in *A. digitata*. The content of soluble dietary fiber in *A. digitata* pulp was significantly higher than in the other fruit pulps.

**Table 1:** Insoluble and soluble dietary fiber content expressed on the basis of dry matter (n=3)*

Fruit part, Location_year	Dry matter	Insoluble dietary fiber (g/100 g)	Soluble dietary fiber
*Adansonia digitata* pulp			
Tete_2008	86.4±0.5	14.2±0.4	60.3±1.9
Tete_2009	86.5±0.1	14.7±0.0	65.6±0.6
Vilankulos_2009	85.6±0.5	16.1±0.8	57.3±0.3
*Adansonia digitata* kernels			
Tete_2008	92.7±0.3	17.4±3.4	14.0±0.6
Tete_2009	91.7±0.1	20.9±1.3	17.0±3.4
Vilankulos_2009	90.1±0.1	14.7±0.0	42.6±1.8
*Adansonia digitata* whole seed			
Tete_2009	98.2±0.0	56.5±3.1	15.9±1.5
Vilankulos_2009	96.4±0.0	62.0±1.2	16.3±0.6
*Landolphia kirkii* pulp			
Marracuene_2008	26.9±0.1	3.5±0.6	4.6±0.1
Marracuene_2009	23.9±0.2	4.9±0.8	4.3±0.3
Manhica_2009	20.4±0.2	4.9±0.4	5.8±1.2
*Salacia kraussii* pulp			
Marracuene_2008	16.1±037	3.3±0.1	6.0±0.4
Manhica_2008	14.0±0.7	2.6±1.2	7.6±1.0
Manhica_2009	16.5±0.2	5.5±0.3	7.1±0.4
*Sclerocarya birrea* pulp			
Manhica 2009	16.7±0.0	7.7±1.7	10.5±0.9
*Sclerocarya birrea* kernels			
Manhiça 2008	93.6±0.2	17.6±2.9	10.5±2.7
Manhiça 2009	95.0±0.0	18.5±1.5	8.4±2.4
*Vangueria infausta* pulp			
Marracuene_2008	34.9±0.2	45.6±1.4	24.3±1.4
Marracuene_2009	30.0±0.1	45.8±0.9	26.3±0.9
Manhica_2008	27.9±0.2	41.0±0.8	10.6±0.8
Manhica_2009	34.5±0.7	30.9±1.9	23.1±1.9

* ± indicates standard deviation.

**Figure 1:**
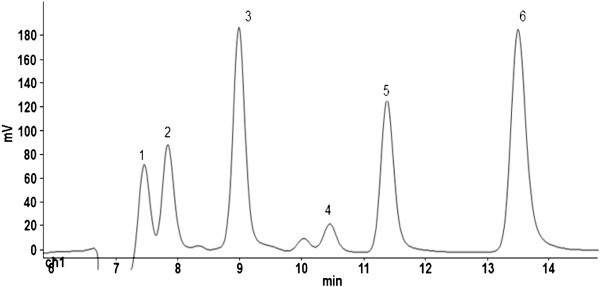
**Chromatogram from the analysis of organic acids in*Salacia kraussii.*** The peaks correspond to 1- citric acid, 2- tartaric acid, 3- malic acid, 4- succinic acid, 5 and 6 unknowns.

A chromatogram from the analysis of organic acids in *S. kraussii* is shown in Figure 1. The peaks corresponding to the different acids are indicated by numbers. The calibration curve was linear within the concentration ranges used for the analysis.

The results of the determination of organic acid content based on wet weight are shown in Table 2. Fruits collected in Manhiça, 2009 were used for this analysis. Citric acid was found in all fruits, with the highest contents in *A. digitata* (25.7 g/kg) and *L. kirkii* (21.5 g/kg). Malic acid was detected at concentrations between 0.4 g/kg and 2.1 g/kg. Succinic acid was found at concentrations of 0.1 g/kg or below. Tartaric acid was detected at trace levels only in *S. birrea*.

**Table 2:** Organic acid content in fruit pulp expressed on the basis of wet weight (n = 3)*

Sample	Citric acid	Tartaric acid	Malic acid (g/kg)	Succininc acid
*Adansonia digitata*	25.7±2.1^*^	ND	1.6±0.2	0.1±0.0
*Landolphia kirkii*	21.5±2.1	ND	0.4±0.1	0.03±0.0
*Salacia kraussii*	0.9±0.3	ND	1.1±0.1	0.1±0.0
*Sclerocarya birrea*	8.5±1.3	trace	1.2±0.2	0.1±0.0
*Vangueria infausta*	6.2±0.5	ND	2.1±0.2	0.1±0.0

* ± indicates standard deviation, ND= not detected.

The mineral contents in the fruit pulps, kernels and seeds, expressed as mg/100 g dry matter, are presented in Table 3. The relative standard deviation of the mean values was generally below 5%. The content in different fruit samples varied considerably, from undetectable levels of selenium up to 2753 mg/100 g for potassium (*S. birrea* pulp). The richest source of calcium was *A. digitata* both for pulp (308 to 366 mg/100 g) and for kernels (293 to 347 mg/100 g.) The iron content in the pulps and kernels generally ranged from 1.0 - 4.0 mg/100 g, while *S. kraussii* pulp contained 9.0 mg/100 g, and the whole seeds of *A. digitata* contained 29 mg /100 g. The highest content of magnesium was found in the kernels of *A. digitata* (626 to 706 mg/100 g) and *S. birrea* (396 – 436 mg/100 g). Selenium was not analyzed in all fruits, but was detected in *V. infausta* (1.5 mg/100 g). The richest source of zinc was the kernels of *A. digitata* (5.2 – 5.7 mg/100 g) and *S. birrea* (4.5 mg/100 g).

**Table 3:** **Mineral content in the pulps, kernels and whole seeds of the studied wild fruits in Mozambique**

Fruit part, Location_year	Dry matter g/100 g	Ca	K	Fe	Mg	Na mg/100 g	P	S	Se	Zn
*Adansonia digitata* pulp										
Tete_2008	90.1	326	2308	2.0	162	5.0	40	51	nd	-
Tete_2009	86.4	308	2392	2.0	129	2.0	40	41	-	0.5
Vilankulos_2009	86.5	366	2360	1.0	102	5.0	30	43	-	0.6
*Adansonia digitata* kernels										
Tete_2009	92.2	293	1416	6.0	626	1.0	1229	263	-	5.7
Vilankulos_2009	90.6	347	1451	4.0	706	2.0	1518	269	-	5.2
*Adansonia digitata* whole seed										
Tete_2008	93.6	220	1238	29	360	2.0	523	131	nd	-
*Landolphia kirkii* pulp										
Manhiça 2009	20.4	28	1840	4.0	51	21	55	20	-	1.4
*Salacia kraussii* pulp										
Manhiça 2009	8.9	127	2056	9.0	207	35	153	191	-	0.6
*Sclerocarya birrea* pulp										
Manhiça 2009	15.9	201	2753	3.0	138	30	178	90	-	.0.7
*Sclerocarya birrea* kernels										
Manhiça 2008	92.7	60	622	4.0	436	7.0	871	284	nd	-
Manhiça 2009	95.0	81	531	4.0	396	6.0	719	269	-	4.5
*Vangueria infausta* pulp										
Manhiça 2009	34.4	90	1249	3.0	65	18	92	45	2.0	-

The relative standard deviation was in general < 5%, nd = Not detectable; - = not analyzed.

## Discussion

The literature provides little data on dietary fiber in the fruits investigated and we have found no data on soluble or insoluble dietary fiber. Our data thus contributes substantially to knowledge about dietary fiber content in wild fruits. The analyses conducted showed that all fruits selected for the study contained both soluble and insoluble dietary fiber, and the total amount of dietary fiber in the pulps ranged from around 10 to 80 g/100 g dry matter (Table 1), thus indicating their potential for improving nutritional content in the local diet.

The largest amount of dietary fiber was found in *A. digitata* pulp. The amount 80.3 g/100 g was much higher than in previous studies, which ranged from 5.4 g/100 g in fresh weight to 45 g/100 g in dry weight (Saka and Msonthi 1994; Lockett et al. 2000; Murray et al. 2001; Osman 2004). The high content of dietary fiber in the present study, of which 80% was soluble dietary fiber, could thus provide a substantial contribution to total dietary intake. For example, the consumption of 20 g *A. digitata* pulp can supply 42 – 52% of the recommended daily intake (RDI) for children from 4 to 13 years of age and also for pregnant women (National Research Council 2005). The high content of soluble dietary fiber could also help to control serum cholesterol levels, reduce other risk factors for cardiovascular disease (Van Duyn and Pivonka 2000), and reduce appetite and caloric intake (Anderson et al. 1994).

*V. infausta* pulp also contained large amounts of total dietary fiber (on average 62 g/100 g), of which more than half was insoluble dietary fiber. Several studies have shown that intake of fruits rich in insoluble dietary fiber benefits weight control and health of the large intestine; the insoluble fiber decreases intestinal transit time and increases fecal bulk (Al-Farsi et al. 2005; Hamilton et al. 1992). Consumption of 100 g *V. infausta* pulp can supply up to 40% of RDI for children and pregnant women (National Research Council 2005). In a report on dietary fiber in *V. infausta*, the content of acid detergent lignin was reported to be 35.5% (fresh weight), acid detergent fiber 39.5% and neutral detergent fiber 39.4% (Amarteifio and Mosase 2006). The discrepancies between our findings and previous research may be due to the difficulty in comparing data on dietary fiber when it isn’t clear whether figures refer to dry or fresh weight. Furthermore, different analytical methods may have been used in different studies, including different types of dietary fiber.

Our data on the amounts of dietary fiber in *S. birrea* pulp were about half of that found in another study determining dietary fiber after extraction of fat by a gravimetric method, 37.7 g/100 g (Murray et al. 2001). In a report where AOAC method was used, the amount of acid detergent lignin was 13.7% (fresh weight), acid detergent fiber 16.3% and neutral detergent fiber 16.1% (Amarteifio and Mosase 2006).

The total amount of dietary fiber in *L. kirkii* pulp was low (10 g/100 g) compared with that of the other fruits in this study, but very similar to that of *L. oweriensis*, of the same family (Effiong and Udo 2010). The amount of dietary fiber in *S. kraussii* pulp was similar to *L. kirkii*. The amounts of dietary fiber in *S. krausii*, and *L. kirkii* pulps are in the same range as in for example avocado, 6.7 g/100 g, and guava, 12.7 g/100 g (Li et al. 2002).

The whole seeds of *A. digitata* contained large amounts of insoluble dietary fiber. These seeds are often crushed into a powder in rural cooking and mixed with other ingredients to make a sauce. Increased use of this local custom may thus help to increase the intake of dietary fiber.

In the kernels of *A. digitata* the total content of dietary fiber was on average 42.2 g/100 g, which is higher than that found in other studies (Murray et al. 2001). The kernels of *S. birrea* contained similar amounts of insoluble dietary fiber as *A. digitata* kernels, but the amount of soluble dietary fiber was lower. There is no report in the literature about dietary fiber of *S. birrea* kernels. However, in cashew nuts and peanuts, which are commonly consumed Mozambique, the total dietary fiber content was 3.9 g/100 g for cashew nuts and 5.2 g/100 g for peanuts in fresh weight (de Oliveira Sousa et al. 2011).

The effects of dietary fiber cannot, however, be considered in isolation. Although dietary fiber provides many health benefits, it may affect mineral absorption negatively due to the capacity of the fibers to bind cations (López and Martos 2004). The ability of dietary fiber to interfere with iron absorption is especially negative for human nutrition (Reinhold et al. 1981). However, citric and malic acids in fruits promote iron absorption (Gillooly et al. 1983), and improve iron solubilization (López and Martos 2004).

Fortunately, citric and malic acid were found in all fruits in this study (Table 2). Citric acid ranged from 0.9 g/kg of wet weight basis in *S. Kraussii* to more than 20 g/kg in *A. digitata* and *L. kirkii.* For malic acid the results were lower and ranged from 0.4 g/kg to 2.1 g/kg. Succinic acid was found in low amounts in all fruits, around 0.1 g/kg, and tartaric acid was detected in trace levels in *S. birrea*. No data in the literature are available on organic acids in the selected wild fruits; however, one type of wild fruit called medlar (*Mespilus germanica L.)* was reported to contain around 4 g/kg fresh weight of citric acid and malic acid (Glew et al. 2003). For comparison, the citric acid concentrations in some traditional fruits are: pineapple (2.2 g/kg), orange (4.5 g/kg), grapes (13.1 g/kg) and lime (41.2 g/kg) (Falade et al. 2003).

Although there were large variations in the mineral content in the different fruits (Table 3), we did not observe any pronounced difference between growth place or harvest year, and the small differences found may be explained by soil, climate and weather conditions.

The highest amounts of calcium from the fruits selected in the present study were found in *A. digitata* pulp and kernel, around 300 mg/100 g dry matter. The amounts are at the same levels as in other reports (Osman 2004; Glew et al. 1997) but higher than in some studies (Saka and Msonthi 1994; Lockett et al. 2000; Amarteifio and Mosase 2006; Eromosele et al. 1991; Sena et al. 1998). *S. birrea* pulp contained 201 mg/100 g, which is about half the amount reported in the literature (Glew et al. 1997), but higher than that in other reports (Amarteifio and Mosase 2006; Eromosele et al. 1991). Calcium is an important factor in bone health, and a high intake is recommended particularly during pregnancy and infancy (Insel et al. 2011) calcium together with phosphor, magnesium and potassium is important for growth and maintenance of bone, teeth and muscle (Insel et al. 2011) and bone metabolism (Ilich et al. 2003; New 2003; Bonjour et al. 2009).

High amounts of phosphor were found in the kernels: *A. digitata* kernels contained up to 1500 mg/100 g, which is four times higher than that in another study (Nnam and Obiakor 2003). Regarding the pulp, there are reports showing higher phosphor content than in our study, for example 452 mg/100 g in *A. digitata* (Sena et al. 1998) and 264 mg/100 g in *S. birrea* (Glew et al. 1997).

High content of potassium, more than 2000 mg/100 g, was found in pulps from *A. digitata, S. kraussii* and *S. birrea*. For *A. digitata*, this is in agreement with results from other studies (Saka and Msonthi 1994; Amarteifio and Mosase 2006), while some reports show lower values. For *S. birrea*, our data agree with other results (Amarteifio and Mosase 2006). The content of potassium in *V. infausta* pulp was on the same level as in one report (Amarteifio and Mosase 2006) but almost 7 times greater than in another report (Saka and Msonthi 1994). The potassium content in kernels of *S. birrea* was somewhat higher than that in another report (Glew et al. 2004). In the whole seeds of *A. digitata,* potassium is higher than in other reports (Osman 2004; Nkafamiya et al. 2007). Potassium, together with sodium, regulates muscle contraction and nerve impulse transmission, and a high potassium/sodium ratio may assist the excretion of excessive salt and water (Arthey et al. 2001). All fruits in our study had low amounts of sodium and thus the potassium/sodium ratio was high in the fruits.

The whole seeds of *A. digitata* had extremely high iron content, 29 mg/100 g, while data in the literature range from 1.83 to 6.36 mg /100 g (Lockett et al. 2000; Osman 2004; Glew et al. 1997; Nkafamiya et al. 2007). *S. kraussii* had the highest iron content of the pulp, 9 mg/100 g. In the other pulps it was 2 to 4 mg/100 g. In the literature, the amounts of iron in *A. digitata* ranged from 0.1 to 9.3 mg/100 g (Saka and Msonthi 1994; Lockett et al. 2000; Amarteifio and Mosase 2006; Glew et al. 1997; Eromosele et al. 1991; Sena et al. 1998; Glew et al. 2004) and in *S. birrea* from 0.07 to 2.49 mg/100 g (Amarteifio and Mosase 2006; Glew et al. 1997; Eromosele et al. 1991). For *V. infausta* there is one report showing 0.09 mg /100 g (Amarteifio and Mosase 2006) and one showing 28.3 mg/100 g (Saka and Msonthi 1994). Very high iron content, 129 mg/100 g dry matter, was reported in *L. oweriensis*, which is a fruit in the same family as *L. kirkii* (Effiong and Udo 2010). Iron is necessary for the transport of oxygen in the blood to the cells and for supplying the body with energy, for immune function and nerve health and in addition, it is a cofactor in numerous reactions (Insel et al. 2011).

The kernels of *A. digitata* had the highest magnesium content, around 600 – 700 mg/100 g. This is higher than the magnesium content in other kernels or seeds. For example dried pumpkin seeds contain 540 mg/100 g, linseed 392 mg/100 g and sunflower seeds 355 mg/100 g (National Food Agency 2012). Our data on whole seeds are at the same level as in other reports for *A. digitata* (Lockett et al. 2000; Osman 2004; Glew et al. 1997) and for *S. birrea* (Glew et al. 2004). Magnesium is of great importance for cardiac and nerve function, is involved in more than 300 biochemical reactions in the body and is involved in energy metabolism and protein synthesis (Insel et al. 2011).

The zinc content in the kernels of *A. digitata* and *S. birrea* was around 5 mg/100 g, which is in agreement with literature for *S. birrea* (Glew et al. 2004) and higher than previous data for *A. digitata* (Nnam and Obiakor 2003). Zinc increases the affinity of hemoglobin for oxygen, participates in taste perception and interacts with a number of hormones. In addition, the body needs zinc to grow and develop properly during pregnancy, infancy, and childhood (Brown et al. 1999; Insel et al. 2011).

The results of the mineral analysis can be compared with the RDI for children 4–13 years of age and pregnant women 19–30 years of age (National Research Council 2005). For example, 100 g of fresh *A. digitata* pulp can contribute on average 23% of the iron and 30% of the calcium RDI for children (4–13 years) and almost 29% of the calcium for pregnant women. Furthermore, 100 g *S. birrea* pulp can contribute 13% of the magnesium RDI for children (4–13 years) and 33% for pregnant women, and almost 41% of zinc requirements. Consumption of 60 g *A. digitata* kernels can supply around 30% of iron, almost 50% of zinc and more than 100% of magnesium RDI for children.

## Conclusion

New data on dietary fiber, organic acids and mineral content have been obtained for five wild fruits and selected seeds. The highest content of dietary fiber was found in *A. digitata* pulp. It was also found that fresh *A. digitata* pulp can contribute a large amount of the iron and calcium RDI for children and that kernel of *A. digitata* and *S. birrea* can contribute significantly to the magnesium and zinc requirements for pregnant women. Thus some of the fruits and kernels studied show large potential to reduce mineral deficiencies in local diet especially in children. The research results highlight the significance of wild edible fruits as a cheap source of nutrients and the benefits of increasing the use these species as dietary supplements. Initiatives should be put in place to promote consumption and domestication of edible indigenous fruit: to improve the nutritional and health status of women and children, contributing to income generation and stimulating rural economic development.

## Materials and methods

### Fruit samples

Five fruits were used for the analysis: *Adansonia digitata (A. digitata)* (Fam. Bombaceae, local name n‘buvu or malambe), *Landolphia kirkii (L.kirkii)* (Fam. Apocynaceae, local name wungwa), *Salacia kraussii (S. kraussii)* (Fam. Celastraceae, local name phinsha), *Sclerocarya birrea (S. birrea)* (Fam. Anacardeaceae, local name canhi) and *Vangueria infausta (V. infausta)* (Fam. Rubiaceae, local name pfilwa). Approximately 5 kg of each fruits were collected in January to July in 2008 and 2009 in four districts of Mozambique, with the exception of the fruits from *Sclerocarya birrea*, which were collected only in 2009. Unblemished fruits were selected and washed, the skin and seeds were removed and the remaining parts (pulp) were homogenized in a blender to obtain 100 g pulp of each type of fruit. Different numbers of fruit were used, depending on size and mass of pulp. Seeds from *Adansonia digitata* and *Sclerocarya birrea* were crushed and the kernels were removed, milled and sieved. The dry matter content in the pulps was determined immediately. Fruit pulps and kernels for analysis of dietary fibre, organic acids and minerals were vacuum packed in plastic bags and stored at −18°C.

### Chemicals

Chemicals and solvents were of analytical grade. Sulphuric acid, nitric acid, hydrochloric acid, sodium di-hydrogen phosphate, di-sodium hydrogen phosphate; sodium hydroxide, ethanol and acetone were from Fluka (Sigma-Aldrich, Steinheim, Germany). Pepsin (2000FIP U/g) obtained from Merck (Darmstadt, Germany), pancreatin from Fluka (Sigma-Aldrich, Steinheim, Germany), and celite were used for the analysis of dietary fibre. Lithium chloride, organic acid standards (citric, malic, tartaric and succinic) were from Merck (Darmstadt, Germany).

### Analysis

#### Dry matter

To determine the dry matter content, 2 g samples were dried in an oven at 105°C until constant weight (AOAC 2000 method 920.151). The samples were weighed before and after drying and the contents of dry matter were calculated. All determinations were performed in triplicate.

#### Dietary fiber

The content of total dietary fiber was first analyzed using an enzymatic gravimetric method, and then divided into fractions of either soluble dietary fiber (SDF) or insoluble dietary fiber (IDF) (Asp et al. 1983). All experiments were performed in triplicate. The sample (0.5-1.0 g) was suspended in a phosphate buffer and hydrochloric acid was added to adjust the pH to 1.5. The sample was digested by pepsin for 60 min at +4°C and then the pH was adjusted to 6.8. Pancreatin was added and the sample was incubated for 60 minutes at 40°C and the pH was adjusted to 4.5. The solution was filtered and the insoluble residue was washed with distilled water, 95% and 99% of ethanol and then dried overnight at 105°C, cooled and weighed (IDF). The filtrate was precipitated with hot 95% ethanol and filtered, washed with ethanol (78%, 95% and 99%), dried, cooled and weighed (SDF). The results were corrected for protein and ash contents.

#### Organic acids

Citric, malic, succinic and tartaric acids were analysed using ion exchange chromatography (Metrohm International, CH-9101, Herisau, Switzerland) with inverse suppression and conductivity detection. Reference samples of the organic acids (Merck, Darmstadt, Germany) were injected via a 20 μl loop onto a column (250 mm × 7.8 mm, 9 μm, polystyrene/divinylbenzene copolymer, Metrosep 6.1005.200, Switzerland) and eluted isocratically at a flow rate of 0.5 mL min-1 and a pressure 3.8 MPa. The suppressor system was regenerated by pumping a solution of 10 mM LiCl, together with Millipore water, through the system. Different ratios of the mobile phase (0.5 mM sulphuric acid and acetone) were tested, as well as different column temperatures (30, 40 and 50°C). The optimal conditions for separation of the organic acids were 0.5 mM sulphuric acid and acetone (85:15, v/v) and 30°C. Stock standard solutions (1 mg/mL) of the organic acids were prepared and kept at +4°C. Different dilutions of the stock solutions were used for the calibration curves. Fruits collected in 2009 in Manhiça were used for this analysis. About 500 mg fruit pulps were mixed with 5 ml sulphuric acid (Ultra Turrax TP18/10) for 2 minutes and then centrifuged at 2000 rpm for 15 minutes. The supernatants were filtered through 0.45 μm membrane filters and diluted with Millipore water to appropriate concentrations. The retention time and peak areas were compared with reference samples run under the same conditions and used to calculate the concentration of the organic acids in the fruit samples. All experiments were performed at least in triplicates.

#### Minerals

Selected samples of the pulps, seeds and kernels were prepared for determination of mineral content. The samples were digested in a microwave oven with concentrated nitric acid and then analysed by Inductively Coupled Plasma-Atomic Emission Spectrometry (ICP-AES, Perkin Elmer, OPTIMA 3000 DV). The experiments were performed in duplicate.

### Statistical analyses

The results of the dietary fibre were subjected to analysis of variance. Differences between means were tested at 5% probability by Turkey’s test, using SPSS program (version 13).
